# Advancing the Population Needs-Based Health Workforce Planning Methodology: A Simulation Tool for Country Application

**DOI:** 10.3390/ijerph18042113

**Published:** 2021-02-22

**Authors:** James Avoka Asamani, Christmal Dela Christmals, Gerda Marie Reitsma

**Affiliations:** 1 Centre for Health Professions Education, Faculty of Health Sciences, North-West University, Potchefstroom Campus, Building PC-G16, Office 101,11 Hoffman St., Potchefstroom 2520, South Africa; asamanij@who.int; 2 Intercountry Support Team for Eastern and Southern Africa, Health Workforce Unit, Regional Office for Africa, World Health Organisation, 82-86 Cnr Enterprise/Glenara Roads, Harare CY 348, Zimbabwe; gerda.reitsma@nwu.ac.za

**Keywords:** population health needs, needs-based health workforce, health workforce planning, health service planning, human resources for health, workforce modelling, need-based modelling

## Abstract

Although the conceptual underpinnings of needs-based health workforce planning have developed over the last two decades, lingering gaps in empirical models and lack of open access tools have partly constrained its uptake in health workforce planning processes in countries. This paper presents an advanced empirical framework for the need-based approach to health workforce planning with an open-access simulation tool in Microsoft^®^ Excel to facilitate real-life health workforce planning in countries. Two fundamental mathematical models are used to quantify the supply of, and need for, health professionals, respectively. The supply-side model is based on a stock-and-flow process, and the need-side model extents a previously published analytical frameworks using the population health needs-based approach. We integrate the supply and need analyses by comparing them to establish the gaps in both absolute and relative terms, and then explore their cost implications for health workforce policy and strategy. To illustrate its use, the model was used to simulate a real-life example using midwives and obstetricians/gynaecologists in the context of maternal and new-born care in Ghana. Sensitivity analysis showed that if a constant level of health was assumed (as in previous works), the need for health professionals could have been underestimated in the long-term. Towards universal health coverage, the findings reveal a need to adopt the need-based approach for HWF planning and to adjust HWF supply in line with population health needs.

## 1. Introduction

The attainment of health objectives, including universal health coverage (UHC), requires a responsive health system underpinned by an adequate number of multidisciplinary health professionals with an appropriate skills mix, trained, motivated, committed, and supported to perform [1,2]. However, a looming global health workforce (HWF) shortage of 18 million by 2030, predominantly in low- and middle-income countries [1] is a serious threat. The deficit is traced to inadequate production capacity on the part of health professions education institutions, coupled with budgetary restrictions for employment and rapid attrition of existing health workers through migration and retirements [3,4,5,6]. It is thus estimated that more than half of some US$3.9 trillion needed in health investments by 2030 must be in HWF education and employment [7]. Although this demonstrates the centrality of the HWF in global health goals, chronic underinvestment continues to be a drawback, and it is linked to defective and ad-hoc HWF planning [1,8,9], which is a part of, and cannot be delinked from, the overall health service planning [10].

The HWF component of health service planning is a complex, multi-sectoral and multifaceted process aimed at achieving an optimal balance between the supply of the different categories of health professionals (in the right mix) and the optimal need and demand for such professionals in both the short and long-term [11]. Roberfroid et al. [12] outlined three aspects of analyses that inform HWF policy and plans: supply analysis, needs (and demand) analysis, and gap analysis [12]. Besides, understanding the resource implications (in terms of cost) of the HWF supply and need viz-a-viz the available and anticipated budget (or fiscal space) is critical for policy and strategic decisions within the broader context of health service planning.

The supply-side analysis of the HWF planning involves taking stock of the available health professionals, and determining (or forecasting) the future inflows and outflows [13,14]. A careful application of the stock-and-flow approach has shown promise in modelling the supply of health workers [15,16] but will mostly be dependent on the assumptions, accuracy, and relevance of the input data.

The second part of health workforce planning involves analysing the required calibre and number of health professionals in a specific jurisdiction necessary to meet its health system objectives [14,17]. There are several approaches with varied conceptual underpinnings for this analysis. Some are based on the observed levels of health service utilisation (service demand); health services targets; number and size of health facilities (health facilities staffing norms); simple population ratios; and population health needs (or epidemiological approach). Several texts examine each of these approaches in depth [see, for example, [14,18,19,20,21,22,23,24]]. Although each method has its merits and downsides, and none has been explicitly shown to be superior in all contexts, the needs-based approach to health workforce planning has a strong instinctive appeal in line with the UHC efforts. For instance, the World Health Assembly (WHA) adopted resolution WHA69.19 in 2016 which profoundly urged countries to make a paradigm shift in health workforce planning toward matching ‘…the supply of health workers to population needs, now and in the future’ [1].

The needs-based approach makes ‘… explicit consideration of population health needs [using] direct measures of levels of health [or health status] that give rise to need for care—and the planned [or otherwise needed] number and type of services to be provided to address those needs’ [25]. To apply this approach, one has to ‘combine information on the health status of the population with disease prevalence, demographics and appropriate standards of care [within the jurisdiction]’ [12]. Even though the theoretical foundations of the needs-based approach have been well advanced and empirically tested in different settings [26,27,28,29,30,31], its applications for policy and planning in countries are still limited [32] even in high-income countries [17]. This is not only blamed on the intensity of data requirements [33] but also the lack of ready-for-use, open-access models or software for use by health planners and human resource practitioners.

This paper presents an open-access, ready-to-use needs-based HWF planning model that integrates supply analysis with needs-based requirements to address methodological gaps and enhance the uptake of the needs-based approach planning in countries and their cost implications for training and employment viz-a-viz available budgets. To illustrate its value, we applied the model for midwives and obstetrician/gynaecologist in the context of maternal and new-born care in Ghana. The rest of the paper is divided into a conceptual framework; an empirical framework; results of an applied example; discussion; and conclusion.

## 2. Conceptual Framework

The need for health professionals is derived from the ‘need for health services’, not for its sake but as a means to remain healthy [14,34]. Thus, it can be assumed that the population in any jurisdiction has a specific need for health services whether or not they have demanded it and can afford it [14]. Therefore, to estimate the optimal need for health professionals, one would, first have to model the population’s need for health services [28]. Building on the conceptual framework proposed by Birch and colleagues [35], the population’s need for health services is a function of three main drivers: (a) The size of the population and its demographic characteristics; (b) the state of health or level of health of the population; and (c) the level of services (type and frequency) that is planned or otherwise necessary to attain and maintain optimal health by the population. The interaction between these variables determines the population’s ‘Need for Health Services’.

The model presented in this paper differs from other models on several levels. Whereas previous models implicitly used a constant rate of disease prevalence (level of health) from the baseline to extrapolate into the future, this model makes adjustments for the expected rate of change over time in the population’s level of health. In one review, Murphy et al. [17] noted that there were ‘…no documents [health workforce plans] that modelled the impacts of changes in [population] needs and potential associated changes … in terms of their impacts on HRH requirements’ (p. 6). This represents a significant limitation in previous works which this model attempts to address in determining the future need for health services and ultimately the needs-based health workforce requirements. The need for health services can then be translated into the health workforce requirements if the category of health professional competent to deliver the service is identified [30] with clear work division [29], and a measure of their productivity is established [27,28].

Where previous needs-based models incorporated productivity [26,29,30,31,33,36], this model defines the productivity measure in terms of a ‘standard workload’ which is the amount of work within one health service activity that a health professional (of a particular type who is competent and well-trained relative to the job specifications) may be able to perform within a given year [37]. The standard workload is, in turn, a function of the available working time of the health professional and the service standard (the amount of time necessary to deliver the given service task within professionally acceptable standards in the jurisdiction or based on service delivery protocols). When the need for health services (the evidence-based health service requirement) is compared with (divided by) the standard workload, the need-based health workforce requirements is established but only for services that can be counted or measured per person. To account for the support or catalytic activities that are undertaken by health workers to enhance the delivery of direct patient/person care, the estimated requirement has to be adjusted for these activities.

On the supply side, for any type of health worker, the model starts by taking into account the annual enrolments of students into health professions training institutions. The total annual enrolment is then adjusted for the average rate of dropouts from each cohort of in-take. The aggregate output from the education pipeline (the number of those who pass successfully through the health professions training institutions) together with those who may migrate into the jurisdiction is adjusted by the average pass rate of any mandatory licensing examination that they may have to sit to make them eligible to practice. This represents the anticipated annual inflow into the existing stock of health professionals (the active workforce stock). The model recognises that not all the active stock (those qualified and with a valid license to practice) will find or take up jobs at frontline service delivery areas. Hence, the active stock is further adjusted by a rate of participation (the proportion of the active health workforce that are practising) to derive the effective supply (practising stock) of the health workforce.

**Figure 1:**
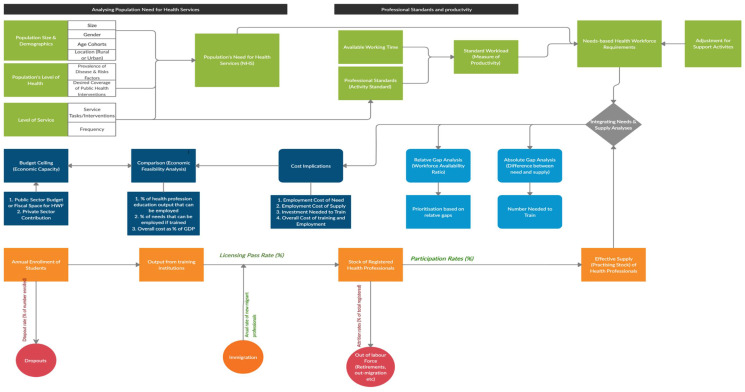
A conceptual framework for comprehensive needs-based health workforce planning. Source: Author’s adaptation from Tomblin Murphy et al. [25]; Birch et al. [35]; Roberfroid et al. [12]; and MacKenzie et al. [29]. Notes: There are different ways people have used the need-based framework—either needs-based demand weighted analysis or full (unweighted) needs-based analysis. Asamani et al. [32] have distinguished these. The present model is for complete (unweighted) needs-based analysis, hence do not make assumptions of weighting needs with demand/utilisation.

The model then compares the needs-based requirement for health professionals with the anticipated supply establishing current and future health workforce gaps [12] in both absolute and relative terms. The cost implications of the supply and the needs-based requirements are computed, which can then be compared with the current and anticipated budgets or other economic indicators. Depending on the resource implications, policymakers can then make adjustments to policies on student enrolment and recruitment, including wages, to improve the supply situation or consider amending service delivery models to adjust the needs-based requirements or a combination of both. Figure 1 illustrates the conceptual framework.

## 3. Materials and Methods

### 3.1. The Empirical Framework

In operationalising the conceptual framework, we present an analytical framework in which we identify four interrelated estimations of the supply of health workers; the need for health workers; gap analysis; and cost implications. Two distinct, fundamental mathematical models have been defined to quantify the supply of, and need for, health professionals, respectively. These are then compared to establish the gaps in both absolute and relative terms as well as the cost to be compared with available (and anticipated) budget or fiscal space—the main driver of how needs are translated into actual demand for health workers.

### 3.2. Modelling the Supply of Health Workforce

The supply of health professionals refers to the pool of qualified health professionals who are willing to find appropriate jobs to offer health services. Thus, the supply (S) depends on the ‘stock’ (T) and ‘flows’ of the health workforce. The stock refers to the current number of the active health workforce, while the flows have two components: inflow and outflow. The inflow represents new entrants to the labour market from domestic training pathways and through immigration, whereas the outflow represents both voluntary exits (loss to other sectors, emigration) and involuntary exits such as retirement, ill health and death [14].

As defined in the National Health Workforce Account (NHWA) [38] adopted by the World Health Assembly in 2017, the stock of any group of health professionals can be categorised into three groups: registered/overall stock; professionally active stock; and the practising stock. The registered/overall stock comprises all those who have registered with the relevant professional regulatory body or authority within a jurisdiction to practice a health profession, irrespective of whether they are within the jurisdiction and practising or not. Within the overall stock (registered), those that maintain good standing with the professional regulatory body by renewing their licenses, are considered the professionally active stock—some of whom may be engaged in other career interests (such as teaching, research and policy) rather than direct health service delivery. Out of the professionally active stock, those that are engaged in (or are willing to find jobs to be engaged in) direct health service delivery are considered the practising stock of health workers. The interest in health service planning is to estimate as close as possible, the current and future stock of the practising health workforce. We modelled the supply using a stock and flow process [29,39] as represented in Figure 1 and illustrated in the following equation:

(1) $$S_{n,t}=\left[T_{n,t-1}\times \left(1-a_{n}\right)+I_{n}\right]\times P$$

○ ***S_n,t_*** is the effective supply of health professional of category ***n***, at time ***t***.
○ ***T_n,t_*** is the overall stock of health professionals (number registered) category ***n*** at time ***t.***
○ ***a_n_*** is the rate of attrition (the proportion of the stock, ***T_n,t-1_*** that died, retired, could not work due to ill-health or migrated out) which adjusts the overall stock to get the professionally active health professional of category ***n***.
○ ***I_n_*** is the inflows of the health professional of category ***n*** from both domestic and foreign sources.
○ **P** is the rate of labour participation which reflects the proportion of the professionally active health professionals that are engaged in direct health service delivery.

Formulae for attrition rate and inflows as defined in equation one is contained in supplementary material 2.

### 3.3. Modelling Population’s Health Need for Health Professionals

Before the model development, we undertook a scoping review of analytical applications of the needs-based approach of health workforce planning [32] in which we synthesised the main considerations towards methodological harmonisation and increased transparency in needs-based health workforce modelling which mostly informed this empirical framework (see Box 1 for a summary of the critical considerations for needs-based health workforce planning synthesised form the scoping review).

Box 1 **Key considerations for comprehensive needs-based health workforce planning. Source: Asamani et al. [32].**

▪ **Defining the scope:**
  ○ Jurisdictional coverage
  ○ The objective of analysis (aligned with health system objective)
  ○ Planning horizon or timeframe
▪ **Analysis of the population health service needs:**
  ○ Population size and demography
  ○ Measures of the population’s level of health (disease burden and health priorities)
  ○ Level of service (identified interventions and their frequencies necessary to address needs)
  ○ Estimating the evidence-based service requirements
▪ **Translating the evidence-based service requirements into health workforce requirement**
  ○ Matching skills and competencies with identified interventions to address needs
  ○ Eliciting standard workload or measures of productivity
▪ **Exploring resource implications:**
  ○ Comparing the needs-based requirements with anticipated supply
  ○ Estimate cost implications viz-a-viz available and anticipated budget
▪ **Conducting sensitivity analyses:**
  ○ Parameter uncertainty (varying input values across their plausible range to explore the impact on the results)
  ○ Structural uncertainty or policy scenarios (varying key assumptions and course of actions to explore the im-pact on the results)
▪ **Conducting model validation:**
  ○ Statistical comparison with previous estimates (if available)
  ○ Stakeholder consultation and feedback

#### 3.3.1. Analysing the Population’s Need for Health Services (Estimating the Evidence-based Service Requirements)

The need for health services is a function of three broad parameters, namely, demographic characteristics of the population (size, gender, age distribution and geographical location); their level of health or health status (disease prevalence and risk factors); and the services planned to address the health deficits or otherwise necessary to maintain optimal health. Building on the works of Birch and colleagues [35] which was further developed by Mackenzie and colleagues [29], we introduce an explicit adjustment for the instantaneous rate of change of the health status or level of health of the population. The relationship between parameters determining the need for health services could then be mathematically expressed as:

(2) $$NHS_{t}=\sum P_{i,j,t,g}\times [H_{h,i,j,t-1}\;\left(1+R_{h}\right)]\times L_{y,h,i,j,t}$$

where:

- *NHS_t_* represents the ‘Needed Health Services’ by a given population under a given service model, $L_{i,j,t}$ over a period of time *t*.
- *P_i,j,g,t_* represents the size of the given population of age cohort *i*, gender *j* in location (rural or urban) *g* at time *t* in a given jurisdiction (this represents the population and its demographic characteristics).
- *H_h,i,j,g,t_* represents the proportion of the given population with health status *h*, of age cohort *i,* gender *j* in location *g* at time *t* (this represents the level of health of the population).
- *L_y,h,i,j,g,t_* represents the frequency of health services of type *y* planned or otherwise required, under a specified service model, to address the needs of individuals of health status *h* among age cohort *i*, gender *j* in location *g* over time *t* (this represents the level of service required by the population).
-
$R_{h}$ is the instantaneous rate of change of the health status, *h* (supplementary material 2 for formula).

Equation (2) represents the evidence-based need for services that address the fundamental equation of how many of the population by age cohort, gender, in a particular location will need a specific type of services? This is vital for any aspect of health service planning—health workforce, essential medicines, infrastructure, or equipment, among others.

#### 3.3.2. Translating the Evidence-Based Health Service Requirements into Health Workforce Requirements

Translating the need for health services or evidence-based service requirements generated from Equation (2) into health workforce requirements is only feasible by making explicit assumptions about some measure(s) of the productivity of health professionals for the specific services that are planned or otherwise required by the population. We define the measure of the productivity of health professionals borrowing the concept of Standard Workload (SW), which underpins the widely used and well-documented Workload Indicators of Staffing Need (WISN) tool developed by the World Health Organisation [37,40,41]. The standard workload is defined as the amount of work of a particular service delivery task that one health professional who is well trained could perform in a year if the health professional dedicated all his/her working to delivering that service [37]. It is a function of two components: (a) The service standard (SS) for the activity to be performed—the average time that a well-trained and motivated health professional will spend to perform the service delivery activity to acceptable professional standards in the context of the jurisdiction; and (b) the available working time (AWT)—the time a health worker was available in one year to do his/her work, taking into account all absences. Equation (3) illustrates the concept of standard workload and supplementary material 2 contains a formula for calculating the available working time.

(3) $$SW_{n,y}=\frac{AWT_{n}}{SS_{y,n}}$$

where:

- *SW_n,y_* is the standard workload for health professional of category *n* when performing health service activity *y*.
- *AWT_n_* is the available working time of the health professional of category *n*
- *SS_y,n_* is the service standard or the time it takes a well-trained health professional of category *n* to deliver the service activity, *y*.

When more than one type of health professional category perform a service delivery task, MacKenzie and colleagues defined a variable for work division [29]. To account for this variable, the estimated need for health services derived in Equation (2) is adjusted for the proportion of work division (which can be represented by *W*) to get the number of service activity, *y* to be performed by a health professional of category *n* for individuals of health status *h*, age group *i*, gender *j* at location *g* over time *t*. The workload division adjusted need for health services can then be divided by the standard workload (defined in Equation (3)), as illustrated in Equation (4).

(4) $$N_{n,t}=\underset{y}{\sum }\frac{\sum (P_{i,j,g,t}\times \left[H_{h,i,j,t-1}\times \left(1+R_{h}\right)\right]\times L_{y,i,j,t})\times W_{y,n,h,i,j,t}}{SW_{n,y}}$$

- *N_n,t_* is the number of health professionals of category *n* required to deliver a given service model *L_y,i,j,_* to a given population over a period of time *t*.
- *W_y,n,h,i,j,t_* is the proportion of services of type *y* to be performed by a health professional of category *n* for individuals of health status *h*, age cohort *i*, and gender *j* at time *t*.

It is worth noting that the standard workload is a measure of productivity relating only to direct patient/client service delivery activities that can be counted per person (or patient). Therefore, the needs-based health workforce requirements estimated using Equation (4) relates mainly to direct person services and excludes indirect patient care or catalytic activities of the health professional.

#### 3.3.3. Adjusting the Health Workforce Requirements for Indirect Patient Care Activities of Health Workers

As demonstrated by Birch et al. [27] and MacKenzie et al. [29], needs-based models recognise that health workers spend some time performing the activities that are essential for supporting the delivery of direct patient/person services, but such activities are not linked to individual patients/clients. While the previous needs-based models accounted for the phenomenon from a supply perspective by defining and adjusting health workforce supply by activity rates (the proportion of health worker’s time spent on direct care) [29], we take the view that for planning, it is appropriate to estimate the number of health professionals needed to cover such support activities that are catalytic for the direct patient care. For example, the process of handing over from one group of nurses in a shift to another group is a crucial component for continuity of care, but because the activity may not necessarily be counted per patient, it could be ignored in needs-based models. Leveraging on the WISN methodology [37], we define a support allowance standard (SAS) as the proportion of a health worker’s time that is spent on the support (or indirect patient care) activities. When the total SAS (in a proportion) is subtracted from the whole, the difference represents a proportion of the health worker’s AWT that is devoted to direct patient/person services [37]. To incorporate the SAS into Equation (4), an adjustment factor known as the support activities adjustment factor (SAAF) is defined—mathematically expressed as the inverse of the proportion of a health professional time left for direct per person care activities [37]. Thus, the overall need-based requirement with both direct and indirect services can be expressed as:

(5) $$N_{n,t}=(\underset{y}{\sum }\frac{\sum (P_{i,j,t}\times \left[H_{h,i,j,t-1}\times \left(1+R_{h}\right)\right]\times L_{y,i,j,t})\times W_{y,n,h,i,j,t}}{SW_{n,y}})\times SAAF$$

where:

- *SAAF* = $\frac{1}{\left(1-\Sigma SAS\right)}$
- *SAS* = proportion of health professional time spent on support activities.

### 3.4. Needs Versus Supply Gap Analysis

The two main quantities estimated in Equations (1) and (5) above (supply and needs-based requirements) can be analytically integrated to compare the current and anticipated gaps in the health workforce in absolute and relative terms.

#### 3.4.1. Establishing the Absolute Gaps

The difference between the projected supply levels and the projected need for a particular health worker category is considered the absolute gap whereby a negative gap is indicative of a supply shortfall, which will be deemed as the number ‘needed to be trained’ by the health professions education institutions [42]. In contrast, a positive gap is indicative of an over-supply from the health professions education institutions in comparison with the need.

(6) $$Absolute\;Gap_{n,t}=S_{n,t}-N_{n,t}$$

where:

- *Absolute Gap_n,t_* is the absolute gap for health professional of type *n* at time *t*.
- *S_n,t_* is the supply of health professional of category *n* at time *t*.
- *N_n,t_* is the needs-based requirements of a health professional of category *n* at time *t*.

#### 3.4.2. Relative Health Workforce Gaps (Staff Availability Ratio, SAR) 

This Is the Ratio of the Needs-Based Health Workforce Requirement That Will Be Met by the Anticipated Supply

(7) $$SAR_{n,t}=\frac{S_{n,t}}{N_{n,t}}$$

The *SAR* shows the anticipated amount of needs-based workload pressure that will be on the current and future health workforce or the proportion of professional standards that can be maintained if interventions are not put in place to influence the supply (and employment) of health professionals. For interpretation, *SAR* of 1 indicates that the anticipated supply will optimally meet the needs-based requirements, whilst a *SAR* of less than one shows that the anticipated supply is failing to meet the needs-based requirements. On the other hand, when the *SAR* is greater than 1, it is indicative of supply outstripping the projected need for health professionals.

### 3.5. Cost Implications for the Estimated Supply and Needs-Based Requirements

To understand the cost implications and investments requirements for employing health workers, the following is conservatively specified:

(8) $$TCS_{n,t}=\sum (S_{n,t}\times K_{n,t})$$

where:

- *TCS_n,t_* is the total wage bill cost of the anticipated supply of health professional category *n* at time point *t*.
- *S_n,t_* is the anticipated supply of health worker category *n* at time point *t*.
- *K_n,t_* is the average income (made up of salaries, allowances and monetary benefits and adjusted for inflation) for health professional of category *n* at time point *t*.

Similarly, assuming there is sufficient supply, the cost implications for filling the needs-based health workforce requirements is conservatively specified as:

(9) $$TCN_{n,t}=\sum (N_{n,t}\times K_{n,t})$$

- *TCN_n,t_* is the total wage bill cost of need-based requirements of a health professional of category *n* at time point *t*.
- *N_n,t_* is the need-based requirements of a health professional of category *n* at time point *t*.
- *K_n,t_* is the average income (made up of salaries, allowances and monetary benefits and adjusted for inflation) for health professional of category *n* at time point *t*.

## 4. An Applied Example for Maternal and New-Born Care

To demonstrate its application and added value, we applied the comprehensive simulation model to estimate the health workforce supply and the requirement for maternal and new-born care in Ghana. The model is designed to allow for disaggregated analysis by sub-national levels (such as regions, states, counties) and differentiation by rural and urban residence. However, for this example, we focused on a composite analysis of the requirements for Ghana. The categories of health workers considered are midwives and obstetricians and gynaecologists because these are the categories of health worker primarily mandated to provide maternal and new-born care in Ghana.

In estimating the needs-based requirements, data on the size and demographic characteristics of the population were obtained from Ghana Statistical Service projections [43]. The maternal and new-born level of health (health status and coverage of essential services) were taken from the 2007 and 2017 Ghana Maternal Health Surveys [44,45] which were compared to establish the instantaneous rate of change. The available working time for the health professionals, the required services to address the gaps in the level of health and the corresponding activity standards, were obtained from the WISN report [46] and Ghana Maternal Health Policy respectively.

For the supply side, the number of students enrolled at health training institutions and pass rates were extracted from the database of the Health Training Institutions Secretariat of the MOH and the professional regulatory bodies (Medical and Dental Council, Nursing and Midwifery Council). Data on the current stock of the health professionals and attrition rate were obtained and triangulated from administrative data of the Ghana Health Service (GHS) [47,48,49], the holistic assessment report of the Ministry of Health [50,51,52,53] as well as the websites of the professional regulatory bodies [54]. See the simulation tool in Microsoft® Excel in supplementary material 1 for the detailed dataset.

## 5. Results

This section briefly describes the excel-based simulation tool based on the model (see supplementary material 1) and the results of the applied example in terms of (1) estimated need-based requirements, (2) estimated supply, (3) gap analyses, (4) cost implications and (5) some sensitivity analyses.

### 5.1. Description of the Simulation Tool in Microsoft Excel

As argued previously by Tomblin Murphy and colleagues [25,55] and recently echoed by MacKenzie et al. [29], needs-based simulation models are not necessarily intended to predict the future but ‘…to integrate the knowledge of different types of HRH (human resources for health) and other aspects of the health care system, such as planned service levels, into a single planning and communication tool to promote understanding of how various factors affect the supply of and requirements for HRH and identify policy levers for influencing these’ (p. 4). The empirical framework was implemented in several linked spreadsheets in a Microsoft^®^ Excel workbook as a deterministic simulation tool (see version 1.2 of the tool as supplementary material 1). The tool is organised into four modules, each with several worksheets that are linked. Modules 1–3 contains the inputs to be entered, while Module 4 contains the outputs or results.

### 5.2. Estimated Needs-Based Requirements and Anticipated Supply

The simulation shows that the needs-based requirement for midwives is about 16,462 in 2020 which will, on average, increase steadily by 3.5% (range: 3.2–4.0%) annually to 23,161 by 2030. By 2025, the needs-based requirement for midwives would increase to 19,409 which represents a 17.9% increase over the estimated requirement in 2020; and a further increase of 19.3% is estimated from the 2025 requirement to reach the 23,161 by 2030. Similarly, the model estimates that 723 obstetricians/gynaecologists are the needs-based requirement in 2020 which will increase at an average rate of 3.4% annually (range: 3.3–3.7%) to about 854 by 2025, an 18% increase from the base requirement in 2020. A further increase of 18.7% from the 2025 estimates is projected for 2030, which brings the total needs-based requirement for obstetricians/gynaecologists to 1013 by 2030.

**Figure 2:**
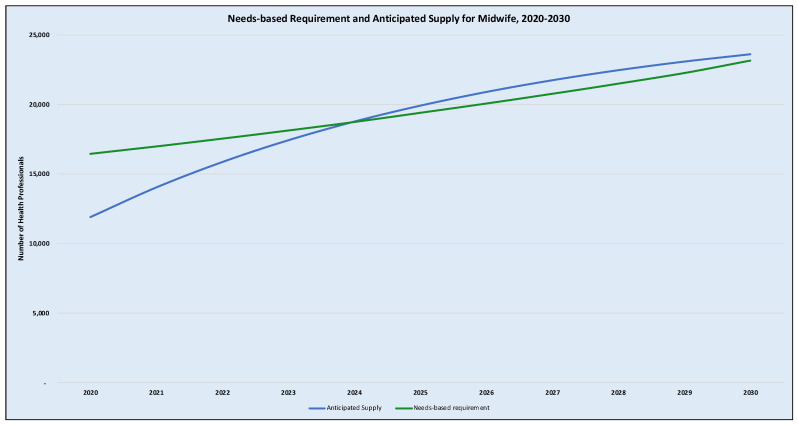
Needs-based requirements and anticipated supply of midwives. Notes: Figure 2 illustrates the projected trend of the need-based requirement for midwives (the green line) as compared to the anticipated trend of supply in the blue line.

**Figure 3:**
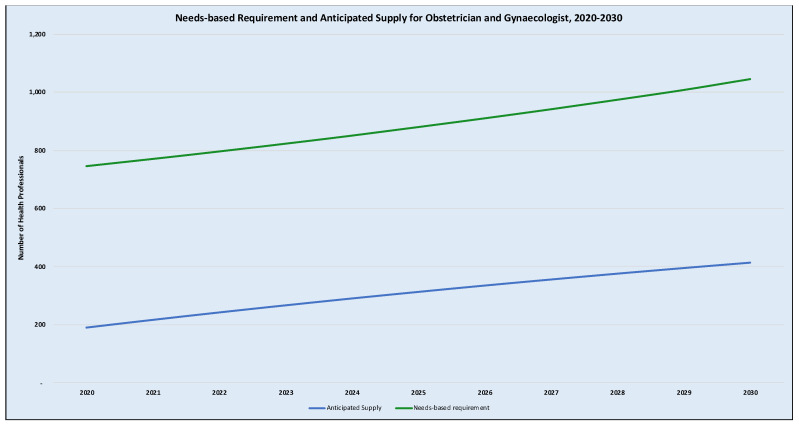
Needs-based requirements and anticipated supply of obstetricians/gynaecologists. Notes: Figure 3 illustrates the projected trend of the need-based requirement for obstetricians/gynaecologists (the green line) as compared to the anticipated trend of supply in the blue line.

At the supply side, the simulation reveals that the existing stock of midwives using a labour participation rate of 93% is about 11,930 in 2020 which could reach about 19,919 (or 67% increase from the baseline) by 2025 and a further 18.5% increase from the 2025 supply levels to 23,602 by 2030. For obstetricians/gynaecologists, they are usually practising general practitioners who undergo specialist training while keeping their jobs; hence they are all placed back to their institutions of employment upon completion. In this context, a 100% labour participation rate was assumed which was the baseline supply in 2020 was 189. It is anticipated that the supply would increase by some 65.7% to 313 in 2025 and a further increase of 32.5% to 415 by 2030. Table 1 and Table 2 show the annual estimates of the needs-based requirement and the anticipated supply, respectively. These estimates are also presented graphically in Figure 2 and Figure 3 as the needs-based requirements versus the anticipated supply for midwives and obstetrician/gynaecologist respectively for the period 2020–2030.

### 5.3. Absolute and Relative Gaps Analyses—Comparing the Needs-Based Requirements and Anticipated Supply

The results of the applied example reveal that initially, the needs-based requirements for midwives outstrip that of the anticipated supply, resulting in a needs-based shortage of 4532 midwives. Comparing the needs-based requirement to the anticipated supply in relative terms, the staff availability ratio, SAR (the proportion of needs-based requirements that is met by the level of supply) for midwives is about 72.5% which imply a shortfall of 27.5% at baseline in 2020. However, an equilibrium is likely to be reached between the supply and needs-based requirement by 2024 if the current trend of production and attrition of midwives continues without further intervention. From 2025, the anticipated supply would then appear to exceed the needs-based requirement marginally, almost at the brink of needs-based oversupply—with SAR of 102.6% by 2025 and a marginal reduction to 101.9% by 2030 (see Table 3).

For obstetricians/gynaecologists, the simulation shows that the needs-based requirement far exceeds that of the anticipated supply throughout the horizon of the projection. As shown in Table 3, the needs-based shortage of obstetrician/gynaecologist specialist is about 534 in 2020, which could reach 599 by 2030 if there is no new intervention to increase the rate of supply or decrease the need or both. The SAR for obstetrician/gynaecologist is a paltry 26.1% in 2020, which is expected to improve to 36.7% by 2025 and 40.9% by 2030. Therefore, under the assumption of maintaining the current rate of production, the model suggests a needs-based shortage of obstetricians/gynaecologists by 74% in 2020 which may only reduce by 15 percentage points to 59% by 2030 (see Table 3).

### 5.4. Implications for Health Professions Education

Assuming that the applied example was a comprehensive analysis, it signals that production/supply of midwives will reach equilibrium with the needs-based by 2024 and after that exceed the needs by roughly 3% by 2025 and then 2% by 2030. Thus, from the health profession education perspective, scaling up the training of midwives may not be warranted beyond the current rate of production. Efforts in the production of midwives could, therefore, be focused on improving the quality rather than further quantitative expansion. On the other hand, the massive needs-based shortage of obstetrician/gynaecologist signals the need to ramp up training by some 599 within the next ten years in addition to the current rate of production. Thus, roughly 60 obstetrician/gynaecologist must be produced per year in addition to the current rate of production. Given that enrolments into specialist medical education such as obstetrician/gynaecologist are drawn from the existing stock of general medical practitioners, it will have a knock-on effect on the need to scale up the training of general practitioners too.

### 5.5. The Cost Implications of the Estimated Needs-Based Requirements and Anticipated Supply

The simulation shows that filling the needs-based requirement for midwives is estimated to cost US$177 million in terms of wage bill which will increase nearly 2.5-folds to US$433.5 million by 2030, due to the expanding needs and adjustments for inflation. The wage bill cost of the anticipated supply of midwives is estimated to be US$118.8 million in 2020 and rising steadily to US$441.7 million by 2030, with the assumption that the rate of labour participation for midwives would remain at 93%. However, if a 100% participation rate is assumed, the wage bill cost of the anticipated supply of midwives could be US$127.7 million in 2020 and gallop to US$653.8 million by 2030.

The needs-based requirement for obstetricians/gynaecologists is estimated to cost about US$14.3 million in terms of salaries, which will gradually increase to US$34.8 million, taking into account both the inflation and expansion in need for obstetricians/gynaecologists. The wage bill cost of the current supply of obstetricians/gynaecologists is estimated to be US$3.5 million in 2020, which may increase by nearly six-folds to US$20.9 million.

The overall need-based requirement for both midwives and obstetrician/gynaecologist is about US$191.3 million in 2020, which may increase to US$309 million by 2025 and US$468.3 million by 2030. By contrast, the aggregate supply of these categories of health workers (midwives and obstetrician/gynaecologist) is estimated to cost US$122.2 million in 2020; US$304.7 million by 2025; and US$462.6 million by 2030 (see Table 4 for details).

### 5.6. Sensitivity Analyses

To explore the impact of the main assumptions made in this model, we varied a number of the variables to document their impact on the output of the model.

***Instantaneous rate of change in the level of health (health status) based on past trends*:** The effect of not including the instantaneous rate of change in the health status indicators (eliminating Equation (5) in the empirical framework) reduced the overall requirements up to 17.4% (n = 3438) for midwives and 23% (n = 189) for obstetricians/gynaecologists by 2030. The SAR for obstetrician/gynaecologist then becomes 28.4% in 2020 (versus 26.1% with the assumption kept) which increases to 50.1% in 2030 (versus 40.9% with the assumption kept). Similarly, the SAR for midwives without the assumption in 2020 is 75.1%, compared with 72.5% when the assumption is kept. Without the assumption, SAR for midwives by 2030 is estimated to be 119.7% as compared to 101.9% when the assumption is kept. From the foregoing, therefore, the further into the future the needs-based projection is made, the more sensitive the results become to the assumption of applying an instantaneous rate of change in the health status of the population.

***Support activities adjustment factor:*** using available data from previous work in Ghana [56], it was estimated in the applied example that two support activities of midwives together consume about 17.5% of their available working time. Similarly, the obstetrician/gynaecologist spends about 6 h per week on clinical meetings and capacity building seminars which represents 15% of the available working time. These translate into support activities adjustment of 21.2% and 17.6% of the needs-based requirements for midwives and obstetrician/gynaecologist, respectively (see Table 5).

**Figure 4:**
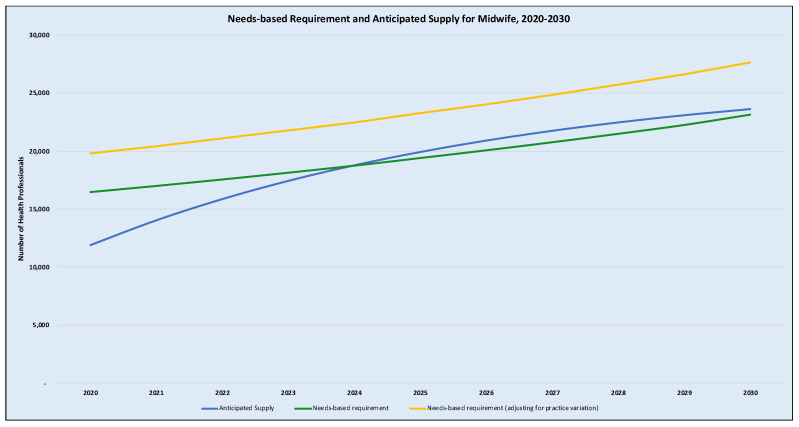
Exploring the effect of variations in activity standards on the needs-based requirements for midwives. Notes: Figure 4 illustrates the impact of practice variation on the projected trend of the need-based requirement of midwives (yellow line) as compared to the original trend (green line) and the anticipated trend of supply (blue line).

***Exploring the impact of practice variations and standard workloads:*** The WISN activity standards in Ghana which was adopted for the applied example had a different set of standard time for accomplishing similar tasks between midwives in primary care health facilities (district hospitals, polyclinics, health centres and CHPS) and secondary/tertiary health facilities. These differences stem from variations in clinical protocols between primary care and tertiary health facilities; and differences in available technology and resources. In the base analysis, the activity standards in the primary care facilities were used since that represents more than 95% of health facilities in Ghana [57]. We explored the potential impact on the model output if the tertiary health facilities standard time (activity standards) were used. As illustrated in Figure 4, the results show that the effect of using the tertiary facilities’ standard time is about 20.3% increase in the needs-based requirements for midwives but weans marginally to 19.3% by 2030. Under this scenario, there will be a needs-based shortage of midwives of 7875 (compared to 4532 in the base case) in 2020. Under this alternative analysis, the staff availability ratio for midwives will be 60.2% in 2020 (versus 72.5% in the base estimation) and 85.4% in 2030 (compared with 101.9% in the base estimation). As shown in previous needs-based models, this finding tends to validate the suggestion that needs-based analysis is highly sensitive to the measures of productivity chosen [25,58], the setting and that of practice variations [27] as well as changing health technology are essential factors to consider in long-term health workforce planning.

## 6. Discussion

The needs-based framework for health workforce planning has been developed and tested by the pioneering scholars since the last two decades and continue to evolve [25,26,27,29,30,31,35,59,60]. Recent advances in the methodology [27,29] have improved its clarity and appeal. Nonetheless, previous long-range projections (dynamic models) made an implicit assumption of monotonously applying baseline levels of health (health status) to future population demographics. Also, previous models did not take into account the support activities undertaken by health workers which are not direct patient care activities but are imperative to facilitate direct patient care. The present model adapted mathematical equations that have been tested over the last two decades for needs-based modelling by incorporating assumptions that were not explicit in previous works to address the aforementioned gaps. In addition, different from previous ones, a measure of productivity was incorporated using the concept of standard workload, which can be directly elicited from health professionals based on the average time they spend in delivering various health service activities. Thus, the model presented in this paper represents a critical and much-needed improvement in the empirical framework for the application of the need-based approach. The World Health Organisation has provided a wealth of description and guidance on the conditions necessary, and how to apply the different HWF modelling approaches [19,24]. However, to the best of our knowledge, there are no dynamic open-access, ready-to-use tools for health planners and human resource practitioners to easily apply the needs-based framework in real-life health workforce planning, especially in the context of low-and-middle-income countries, where the analytical capacity to develop sophisticated models locally is often limited. To bridge this gap and facilitate policy-driven uptake of the needs-based approach to health workforce planning, an easy-to-use, open-access simulation tool developed in Microsoft^®^ Excel has been made available as supplementary material 1.

One fundamental difference between the present model and previous ones is the explicit assumption in the present model that the level of health of the population will likely change, based on previous trends. To account for this assumption, we incorporated an instantaneous rate of change in the population need for health services. Murphy et al. [17] lamented that the needs-based models reviewed failed to take this into account, which represented a significant limitation. Our applied simulation shows that the impact of this assumption on the outcome of needs-based projection is more pronounced, and further into the future the projection is extended. For example, when this assumption was relaxed in our applied example, and a fixed level of health was applied throughout the horizon of the projection (as used in previous models), the needs-based requirements for midwives decreased by up to 17.4%, and that of obstetrician/gynaecologist decreased by up to 23% over the ten years. However, in the base year, the difference was only 3.5% for midwives and 8.8% for obstetrician/gynaecologist. Therefore, not explicitly incorporating the assumption in the empirical framework could result in a substantial underestimation of the actual need for health professionals if longer-term planning is desired. For a one-time point (or static) analysis, the underestimation seems to be minimal and could be within reasonable planning limits, but for dynamic or longer-term analysis, it becomes imperative to take this assumption into account.

Also, the support activities undertaken by health workers were previously not being considered in the estimated need-based requirements in the earlier models. Instead, some models defined a clinical focus term in the supply side of the analysis to adjust the anticipated supply to take into consideration only the proportion of time health professionals spent on direct clinical activities. It is our considered view that the support activities of health professionals are not a supply-side issue, for which reason it ought to be accounted for in the needs-based requirements to enable planning for the numbers needed to cover such support activities. For example, our applied simulation shows that 21% of the estimated requirement for midwives, and 18% for obstetrician/gynaecologist were needed to cover for support activities. Incorporating these assumptions in the needs-based estimates is necessary to inform planning for health professions education and employment to cover for both direct patient/person care and the support activities that catalyse direct care.

To roughly gauge the validity of the model output, we compared the results of the applied example with previous health workforce projections in Ghana [61,62]. Based on health facilities and staffing norms approach [61], it was estimated that Ghana needed 13,554 midwives by 2020 and 18,832 by 2025. Compared to the current estimate of 16,462 midwives needed in 2020 and 19,409 in 2025, the current model estimated 21.45% higher needs in 2020, but the two estimates converge towards 2025 with a variance of only 3.06%. Thus, despite the conceptual differences between the staffing norms and the needs-based approach, the medium-to-longer-term estimates seem to draw a similar conclusion from both approaches in this case of the midwives in Ghana. Furthermore, an administrative analysis of the Ghana Health Service suggested that the production of midwives is at the brink of exceeding the demand [62], whose direction of evidence tends to corroborate with our applied simulation for midwives.

Regarding the scenario for obstetrician/gynaecologists, the applied simulation did not consider other women health issues typically handled in the context of gynaecology, but focused on obstetrics which relates to maternal and new-born care. Thus, the estimates in the applied simulation are by no means the comprehensive needs-based requirements for obstetrician/gynaecologist. Nevertheless, it was previously estimated that Ghana required 750 obstetrician/gynaecologist in 2020 (compared with 723 in the current estimation) and 1044 by 2025 (compared with 854 in the current estimation). Thus, the current estimation is about 3.6% lower in 2020 and 18.2% lower by 2025.

The wage bill in Ghana is centrally managed within the overall personnel emoluments budget of the Ministry of Health. Hence, there is no separate health workforce budget for maternal and new-born health. This made it unfeasible to compare the estimated wage bill cost of the need-based requirements and supply with the available budget that is specific for these categories of health professionals. As part of the application of this model, a comprehensive needs-based health workforce analysis is being undertaken in the context of primary health care in Ghana.

## 7. Limitations

The well-acknowledged limitation of the needs-based workforce planning approach is its relatively intense data requirements [21,29] and lack of end-user tools [32], especially for settings where there is limited analytic capability. The present model partly addresses the latter but also requires extensive data on population size, demographic characteristics, the range of disease burden, health workforce absences and service delivery standards as well as data on the existing stock of health professionals, attrition rates, annual enrolments, pass rates and labour participation rates. Some of these data needs may not be routinely available in many low- and middle-income countries. However, as repeatedly put forward by Tomblin Murphy, Birch and colleagues [17,29,63], an imprecise estimation of health workforce requirements, based on appropriate conceptual underpinnings with the view of progressive improvement in the data adequacy and quality, is more useful than using conceptually invalid models. Cometto and colleagues [64] admonish that health workforce planning is an inexact science; hence the potential impact of data imprecision and uncertainty should always be taken into account. The present simulation model provides an opportunity to explore the impact of data uncertainty on the model needs-based estimates in the form of best and worst cases scenarios. With many countries striving to improve their health workforce data availability and quality through the implementation of the national health workforce account [38,65], the use of needs-based models could become less burdensome and easy to use than first thought.

Also, HWF requirements and supply may not always follow a near process as (implicitly) implied in the mathematical equations since needs depend on disease patterns, health-seeking behaviour of individuals, technological changes/evolution etc., which tend to be non-linear in character. Similarly, the supply side tends to respond to market-related forces, e.g., incentives, levels of pay, working conditions etc., and may not always follow a linear process. Hence, long-range projection with the model should be followed with intermittent revisions based on emerging information and data. In addition, uncertainties such as health emergencies as in the case of COVID-19 pandemic are extremely difficult to be foreseen in models like this. However, as demonstrated by Murphy et al. [33], needs-based models could be adapted for planning in times of health system disruption or emergencies.

Moreover, in the current simulation model, data on indicators for the populations’ health status are required for at least two different time points to enable a calculation of their instantaneous rate of change to operationalise the assumption that the populations’ health needs will be changing in the future based on past trends. This requires the use of consistent sources of data that measure the same indicators periodically, which may become a limitation where such data sources do not exist. In such circumstances, using targets set by local policymakers can be a viable option. Finally, the simulation model is designed on the back of some functions of Microsoft^®^ Excel that were released in October 2020. Hence, those with lower versions may experience compatibility issues.

## 8. Conclusions

The paper builds on the decades of conceptual and empirical work by needs-based pioneering scholars to further advance the needs-based framework for health workforce planning by considering the changing patterns of the population’s level of health (or health status) and support activities performed by health professionals. An open-access simulation tool in Microsoft^®^ Excel has been included with the view of facilitating the use of the needs-based approach for health workforce planning. A real-life example using maternal and new-born care in Ghana is included to demonstrate the value and how the model works. A sensitivity analysis based on the applied example demonstrated that without explicitly incorporating the assumption of a changing future population health status (and need) in the empirical framework could have resulted in a substantial underestimation of the actual need for health professionals in the longer term by up to 17.4% for midwives and 23% for obstetrician/gynaecologist. Also, the simulation results were sensitive to practice variations between primary care facilities and tertiary health facilities by up to 20.3% for midwives. When support activities were considered, the need for midwives and obstetrician/gynaecologist was 21% and 18% respectively higher than if the support activities were not taken into consideration.

## Figures and Tables

**Table 1:** Needs-based requirements for midwives, and obstetricians and gynaecologists.

No.	Health Professionals	Needs-Based Requirements										
		2020	2021	2022	2023	2024	2025	2026	2027	2028	2029	2030
1	Midwife	16,462	16,997	17,556	18,139	18,749	19,409	20,076	20,774	21,504	22,268	23,161
2	Obstetrician/Gynaecologist	723	747	772	798	825	854	883	913	945	977	1013
**Total**		**17,186**	**17,745**	**18,328**	**18,937**	**19,574**	**20,263**	**20,959**	**21,687**	**22,448**	**23,245**	**24,174**

**Table 2:** Estimated supply of midwives, and obstetricians and gynaecologists.

No.	Health Professionals	Estimated Aggregate Supply										
		2020	2021	2022	2023	2024	2025	2026	2027	2028	2029	2030
1	Midwife	11,930	14,057	15,878	17,438	18,775	19,919	20,900	21,739	22,459	23,075	23,602
2	Obstetrician/Gynaecologist	189	216	242	266	290	313	335	356	377	396	415
**Total**		**12,119**	**14,272**	**16,120**	**17,705**	**19,065**	**20,232**	**21,235**	**22,096**	**22,835**	**23,471**	**24,017**

**Table 3:** Gap analysis between needs-based requirements and anticipated supply.

No.	Health Professionals	2020				2025				2030			
		Need (a)	Supply (b)	Gap (b-a)	SAR (b/a)	Need (a)	Supply (b)	Gap (b-a)	SAR (b/a)	Need (a)	Supply (b)	Gap (b-a)	SAR (b/a)
1	Midwife	16,462	11,930	(4532)	72.5%	19,409	19,919	510	102.6%	23,161	23,602	442	101.9%
2	Obstetrician/ Gynaecologist	723	189	(534)	26.1%	854	313	(541)	36.7%	1013	415	(599)	40.9%

SAR: staff availability ratio.

**Table 4:** Cost implications of the needs-based requirements and anticipated supply.

No.	Health Professional	The Estimated Wage Bill in United States Dollars (US$)					
		2020		2025		2030	
		Need	Supply	Need	Supply	Need	Supply
**1**	Midwife	176,988,192.56	118,759,613	285,949,683.80	293,467,630	433,445,908.15	441,711,580
**3**	Obstetrician/Gynaecologist	14,278,392.77	3,454,061	23,090,373.91	11,214,559	34,820,936.99	20,921,755
	**Total**	**191,266,585.33**	**122,213,674.52**	**309,040,058**	**304,682,188**	**468,266,845**	**462,633,335**

**Table 5:** Exploring the effect of incorporating support activities in the needs-based estimation.

No	Health Professional	Intervention	Measurement	Support Allowance Standard (SAS)	Support Allowance Factor (SAAF)	% Adjustment Required to Cater for Support Activities
**1.**	Midwife	Handing over	1 h per day	12.5%	1.212	21.2%
		Clinical/unit meetings	2 h per week	5.0%		
**2.**	Obstetrician/Gynaecologist	Clinical/unit meetings	6 h per week	15.0%	1.212	17.6%

## Data Availability

Data are contained within the article and supplementary material in Microsoft Excel.

## Supplementary Materials

The following are available online at https://www.mdpi.com/1660-4601/18/4/2113/s1. Material 1: Microsoft Excel-based model: population needs-based simulation model for health workforce planning; Material 2: Additional formule.
