# Supplementary material for: Advancing the Population Needs-Based Health Workforce Planning Methodology: A Simulation Tool for Country Application
Source: Int J Environ Res Public Health. 2021 Feb 22;18(4):2113. doi: 10.3390/ijerph18042113 (PMC7926568; doi:10.3390/ijerph18042113)
Supplement: Supplementary file 1 [file ijerph-18-02113-s001.zip › Supplementary material 2.docx]

**Supplementary material 2: Additional formule**

1. **ATTRITION**

The attrition, ***a*** of health professionals of category ***n***, in the analytical model (in equation 1) is defined as:

**… supp. equation 1**

**Where:**

- ***d*** is the number of deaths in health professional of category ***n*** in time ***t-1***
- ***r*** is the number of retirements amongst health worker type n in time ***t-1***
- ***i*** is the number of health professionals of category ***n*** that permanently went out of the labour market due to ill-health in time ***t-1***
- ***e*** is the number of out-migration from the jurisdiction amongst health worker category ***n*** in time ***t-1***

1. **HWF INFLOWS**

The inflows, ***I*** for a health professional of category ***n***, in the analytical model (in equation 1) is also defined as:

**… supp. equation 2**

Where:

- ***I_n_*** is the inflows of health professionals of category ***n***
- ***E_n_*** is the number of enrolments in the health professions education institutions for health professionals of category ***n***.
- ***U_n_*** is the program dropout rate (proportion of a year’s cohort that does not complete the course of training).
- ***Im_n_*** is the number of health professionals of category ***n*** that migrates into the jurisdiction per year.
- ***pr_n_*** is the pass rate of trained health professionals of category ***n*** from the licensing examination.

1. **RATE OF CHANGE IN LEVEL OF HEALTH**

We adopted the standard formulae for instantaneous rate of change [36] which is mathematically expressed as follows:

**… supp. equation 3**

Where:

- ***Ln*** is the natural log
- ***P_h_*** is the proportional change of health status ***h*** over the duration between two timepoint ***d***.
- ***d*** is the duration in years within which the proportional change in health status ***h*** occurred.

1. **AVAILABLE WORKING TIME**

**… supp. equation 4**

- ***AWT_n_*** is the total available working time in a year for a health professional of category ***n***
- ***A*** is the number of possible working days in a year
- ***B*** is the number of public holidays in a year
- ***C*** is the number of annual leave days in a year
- ***D*** is the number to sick leave days entitled by the health professional in a year
- ***E*** is the number of days off due to other leave, such as training in a year.
- ***F*** is the number of working hours in one day.
